# Endothelial dysfunction and cardiovascular risk in post-COVID-19 patients after 6- and 12-months SARS-CoV-2 infection

**DOI:** 10.1007/s15010-024-02173-5

**Published:** 2024-02-07

**Authors:** Paula Poyatos, Neus Luque, Gladis Sabater, Saioa Eizaguirre, Marc Bonnin, Ramon Orriols, Olga Tura-Ceide

**Affiliations:** 1 Department of Pulmonary Medicine, Dr. Josep Trueta University Hospital de Girona, Santa Caterina Hospital de Salt and the Girona Biomedical Research Institute (IDIBGI), 17190 Girona, Spain; 2 Department of Medical Sciences, Faculty of Medicine, University of Girona, Girona, Spain; 3 Biomedical Research Networking Centre on Respiratory Diseases (CIBERES), Madrid, Spain; 4 Department of Pulmonary Medicine, Servei de Pneumologia, Hospital Clínic-Institut d’Investigacions Biomèdiques August Pi I Sunyer (IDIBAPS), University of Barcelona, Villarroel, 170, 08036 Barcelona, Spain

**Keywords:** Endothelial progenitor cell, Post-COVID-19, Vascular injury, Endothelial Biomarkers, Long-COVID, Sequelae

## Abstract

**Introduction:**

SARS-CoV-2 infection causes severe endothelial damage, an essential step for cardiovascular complications. Endothelial-colony forming cells (ECFCs) act as a biomarker of vascular damage but their role in SARS-CoV-2 remain unclear. The aim of this study was to assess whether the number of ECFCs and angiogenic biomarkers remained altered after 6 and 12-months post-infection and whether this imbalance correlated with the presence of long-COVID syndrome and other biological parameters measured.

**Methods:**

Seventy-two patients were recruited at different time-points after overcoming COVID-19 and thirty-one healthy controls. All subjects were matched for age, gender, BMI, and comorbidities. ECFCs were obtained from peripheral blood and cultured with specific conditions.

**Results:**

The results confirm the presence of a long-term sequela in post-COVID-19 patients, with an abnormal increase in ECFC production compared to controls (82.8% vs*.* 48.4%, *P* < 0.01) that is maintained up to 6-months (87.0% vs*.* 48.4%, *P* < 0.01) and 12-months post-infection (85.0% vs. 48.4%, *P* < 0.01). Interestingly, post-COVID-19 patients showed a significant downregulation of angiogenesis-related proteins compared to controls indicating a clear endothelial injury. Troponin, NT-proBNP and ferritin levels, markers of cardiovascular risk and inflammation, remained elevated up to 12-months post-infection. Patients with lower numbers of ECFC exhibited higher levels of inflammatory markers, such as ferritin, suggesting that ECFCs may play a protective role. Additionally, long-COVID syndrome was associated with higher ferritin levels and with female gender.

**Conclusions:**

These findings highlight the presence of vascular sequela that last up to 6- and 12-months post-infection and point out the need for preventive measures and patient follow-up.

**Supplementary Information:**

The online version contains supplementary material available at 10.1007/s15010-024-02173-5.

## Introduction

Coronavirus disease 2019 (COVID-19), caused by the severe acute respiratory syndrome coronavirus 2 (SARS-CoV-2), has represented a major public health crisis [1]. Cellular entry of SARS-CoV-2 involves angiotensin-converting enzyme 2 (ACE2) as receptor and transmembrane protease serine protease 2 (TMPRSS-2) as co-receptor [2]. Although COVID-19 was mainly considered a respiratory disease, it is currently defined as a multiorgan disorder capable of injure many organs [2]. Increasing evidence has shown that SARS-CoV-2 can target the vascular endothelium, triggering an endothelial dysfunction [3]. Hence, COVID-19, is now considered a vascular disease [4, 5]. However, the mechanisms behind SARS-CoV-2 endothelial cell infection are still largely unknown. It is known that under a healthy environment, pulmonary endothelial cells express minimal levels of ACE2. Nevertheless, in COVID-19 patients, the systemic endothelial inflammation increases ACE2 receptor expression in blood vessels, making them more susceptible to SARS-CoV-2 infection [6]. This places the entire vascular system at risk of injury. Additionally, indirect mechanisms such as inflammatory or coagulation activation can also lead to endothelial cell infection and vascular damage [7]. It is likely that both mechanisms occur simultaneously, resulting in endothelial dysfunction and heightened cardiovascular risk.

The endothelium is a dynamic organ responsible for maintaining vascular homeostasis [8]. Disruption of this delicate balance contributes to vascular damage, dysfunction, and the development of various vascular disorders [9]. This imbalance shifts the vascular equilibrium towards vasoconstriction, inflammation, increased permeability, and a pro-coagulant state [10–12].

Several studies to date have shown evidence of an endothelial dysfunction after SARS-CoV-2 infection. Varga et al. [13]*.* found the presence of endotheliitis in several organs of postmortem COVID-19 patients. Rotoli et al. [14]*.* demonstrated that human lung microvascular endothelial cells (HLMVEC) infected with spike protein 1 of SARS-CoV-2 were activated after the infection, increasing the expression of pro-inflammatory mediators and contributing to the development of a pro-coagulative endothelium; and Fogarty et al. [15]*.* showed persistent endotheliopathy in convalescent COVID-19 compared to healthy controls, with an elevation in von Willebrand factor (vWF) and soluble thrombomodulin. Additionally, it is reported that 31% of COVID-19 patients in intensive care unit have suffered thrombotic complications [16].

Diverse biomarkers have been proposed to assess endothelial function in both COVID-19 and post-COVID-19 patients, including flow-mediated dilation (FMD), lung diffusing capacity (DL_CO_) or vascular intima-media thickness (IMT) as non-invasive indicators; and circulating levels of vWF, tissue plasminogen activator (tPA), plasminogen activator inhibitor-1 (PAI-1), soluble thrombomodulin, angiopoietin-2, vascular cell adhesion molecule (VCAM), E selectin, circulating endothelial cells (CECs) or endothelial-colony forming cells (ECFCs) as invasive ones [3, 17]. Recent studies have revealed that many of these biomarkers remain elevated after COVID-19 pathology. Sibila et al. [18] reported that 6-months post-COVID-19 patients with reduced DL_CO_ levels presented higher levels of soluble intercellular adhesion molecule-1 (sICAM-1) and angiopoietin-2, suggesting persistent endothelial activation and damage. Moreover, CECs have been shown to be increased in COVID-19 patients and in COVID-19 convalescents compared to healthy controls [19–21]. In line of these results, we recently reported an abnormal increase in the number of ECFCs in the blood circulation after 3-months of infection compared to healthy controls [21].

Circulating ECFCs are a rare population with a robust proliferative potential that have the ability to form human blood vessels in vivo*,* contributing to neovascularization and re-endothelialization [22, 23]*.* ECFCs are mobilized from the bone marrow or from its niche in the vessel wall in response to ischemia and migrate to sites of vascular damage, promoting vascular regeneration [22, 24]. Changes in ECFCs number and function can be used as biomarkers to evaluate cardiovascular risk and disease progression [25].

Long-COVID, or post-COVID-19 syndrome, refers to a condition characterized by persistent or newly emerging symptoms that endure for more than 12 weeks following the initial infection, and are not attributable to any other specific disease or diagnosis [26]*.*It involves diverse symptoms affecting multiple organ systems, including fatigue, dyspnea, anxiety, and joint pain [27]. Studies show that around 70% of COVID patients experience at least one symptom for months after infection [4, 12]. Recent research by Fogarty et al*.* [15] suggests that persistent endothelial cell activation may contribute to long-COVID pathogenesis.

In the current study, we aim to evaluate whether the number of ECFCs together with angiogenic biomarkers in plasma remain abnormal 6 and 12-months after the infection and whether this alteration correlates with any of the biomarkers and clinical parameters measured or the presence of long-COVID.

## Materials and methods

### Study population

For the study, patients at different time points after overcoming COVID-19, 3 months (*n* = 29), 6 months (*n* = 23) and 12 months (*n* = 20), were evaluated and compared with a group of 31 healthy control subjects (CL). Thirty-five percent of COVID-19 patients at the different times suffered PE during admission, detected by CT examination (Supplementary Fig. S1). All subjects were matched for age, gender, BMI and clinical comorbidities such as hypertension or diabetes. Post-COVID-19 patients included in the study were hospitalized and admitted during the months of March and April 2020, when the pandemic began. Sixty percent of patients were treated in ICU. All subjects were not previously vaccinated and all of them were infected with the same SARS-CoV-2 Wuhan-variant. All patients were discharged from the Pulmonary Medicine Service with severe pneumonia and a diagnosis of COVID-19 by positive PCR. Additionally, COVID-19 patients were subclassified based on whether they had a diagnosis of pulmonary embolism or not. The number of patients at the three time points differs because some patients withdrew from the study. Healthy control subjects were non-hospitalized and non-staff volunteers residing at the same health region as COVID-19 patients. Healthy controls were confirmed negative for SARS-CoV-2 infection at the time of ECFC isolation by PCR and did not suffer any previous COVID-19 infection*.*

The characteristics and comorbidities of patients were collected from their medical records. We evaluated dyspnea according to the modified Medical Research Council scale [28]. We also performed a blood test (including lymphocyte (K/mcL), LDH (mg/dL), ferritin (ng/mL) and troponin T (pg/mL)), pulmonary function tests and a 6-min walking test. Iron (II,III) (µg/dL), transferrin (mg/dL) and NT-proNBP (pg/mL) were measured using the serum fraction. Additional cardiac parameters are shown in the supplementary materials.

### Plasma and serum sample collection

Peripheral blood samples were collected using 8,5 mL red top tubes containing spray-coated silica to aid in clotting and a polymer gel (Becton Dickinson, UK) for serum collection and 10 mL tubes with sodium heparin (Becton Dickinson, UK) for plasma collection. Peripheral blood samples were centrifuged (2000 g, 10 min, RT) to separate the serum and plasma fraction and stored at –80ºC.

### Isolation of ECFC

**Fig. 1:**
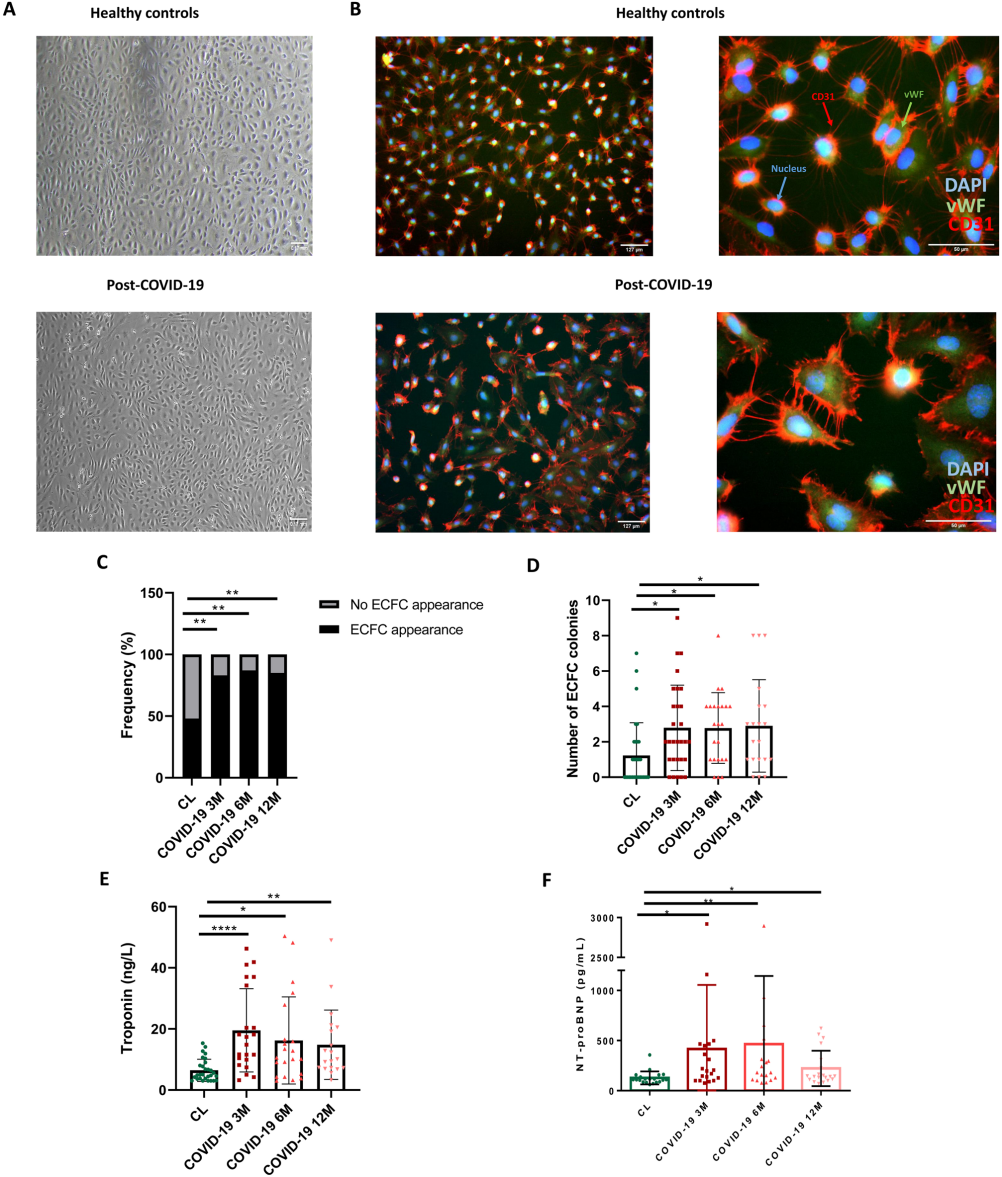
**A** ECFC colonies of CL and post-COVID-19 patients resembling typical cobblestone morphology appeared within 1–3 weeks of culture (4x). **B** Immunofluorescence staining for CD31 (red), vWF (Green), nuclei (blue) of endothelial cells from healthy controls and post-COVID-19 patients (10 ×) (60x). Blue arrows represent the nucleus, green arrows represent vWF and red arrows represent CD31. **C.** Frequency of appearance and no appearance of ECFC colonies in healthy controls and 3, 6 and 12-months post-COVID-19 patients, Chi-square test, **P < 0.01. **D** Number of ECFC colonies in healthy controls and 3, 6 and 12-months post-COVID-19 patients, Kruskal–Wallis test followed by Dunn’s multiple comparisons test, *P < 0.05. **E** Levels of troponin (ng/L) in healthy controls and 3, 6 and 12-months post-COVID-19 patients, Kruskal–Wallis test followed by Dunn’s multiple comparisons test, *P < 0.05, **P < 0.01, ****P < 0.001. **F** NT-proBNP (pg/mL) in healthy controls and 3, 6 and 12-months post-COVID-19 patients, Kruskal–Wallis test followed by Dunn’s multiple comparisons test, *P < 0.05, **P < 0.01. *DAPI* 4′,6-diamidino-2-phenylindole, *vWF* Von Willebrand factor, *CD31* cluster of differentiation 31, *CL* healthy control, *ECFC* endothelial colony-forming cells, *NT-proBNP* N-terminal-pro hormone B-type natriuretic peptide

The isolation of ECFC from all subjects and immunofluorescence analysis were performed as previously described [29, 30] (Fig. 1A, B). Briefly, peripheral blood mononuclear cells (PBMC’s) were isolated by buoyant density centrifugation over Ficoll‐Paque Plus (GE Healthcare), resuspended in endothelial cell medium (ECM‐2 medium, ScienceCell, Research Laboratories) supplemented with 20% fetal bovine serum (FBS hyclone, Cytiva) and 1% penicillin–streptomycin (P/S, Lonza); and plated onto type‐1 rat‐tail collagen‐coated six‐well tissue culture plates (BD Biosciences). Cells were incubated at 37 °C, 5% CO_2_, and 95% relative humidity for 3–4 weeks [30]. The medium was changed every 2 days until the appearance of ECFC colonies. Cells were expanded in ECM-2 culture medium supplemented with 10% FBS and were cryopreserved in 90% FBS with 10% DMSO.

Colonies were counted after appearance, as an association of two or more individual cells together with the presence of a typical cobblestone endothelial morphology of ECFC. These colonies may appear on consecutive days and the number of colonies used in this study is the total number of colonies generated by a subject throughout their culture. The number of days it took for the first colony to appear were also quantified. All these parameters were evaluated between post-COVID-19 patients at the different times after SARS-CoV-2 infection (3, 6 and 12 months) compared to healthy controls and between COVID-19 patients who suffered PE during admission than those that did not. Isolated ECFCs were characterized by nucleocapsid (N) antibody (Anti-SARS-CoV-2 N protein antibody) (Sino Biological). This antibody was utilized in combination with an Alexa Fluor 488-conjugated secondary antibody for immunofluorescence analysis. Additionally, qPCR of NP, ORF1, Rdp and TMRSS2 genes was performed using SYBR Green reagent (Applied Biosystems) (Supplementary Figure S2). Primer sequences are listed in in Supplementary table S1.

### Human NT-proBNP ELISA assay

NT-proBNP protein was measured by Human NT-proBNP ELISA Kit (ab263877) following the manufacturer's instructions. Serum samples of post-COVID-19 patients at the different time points (*n* = 20) and healthy controls (*n* = 20) were assessed. First, standards and samples were added to appropriate wells. Then, antibody cocktail was added to all wells and incubated during 1 h at room temperature. After the incubation, wells were washed three times with wash buffer and incubated during 10 min with TMB development solution. Finally, stop solution was added and plate was read at 450 nm.

### Proteome profiler human angiogenesis array assay

Expression levels of 55 angiogenesis-related proteins spotted in duplicate were assessed by the Proteome Profiler Human Angiogenesis Array (ARY007, R&D Systems, USA) following the manufacturer's instructions. Control plasma samples used were from a pool of 5 different healthy controls from our study population. A pool of 6 patients 3-months post-COVID-19 who had suffered PE, 6 patients 3-months post-COVID-19 who hadn’t suffered PE and another pool of 6 patients 6, 12-months post-COVID-19 after infection were also assessed. All subjects used were matched for age, sex, BMI and comorbidities. Briefly, plasma samples were incubated with an antibody cocktail at room temperature for 1 h. During this incubation, blocking buffer was added to the membrane containing the capture antibodies. After the hour incubation, each mixture was incubated with each washed membrane overnight at 4 °C. The membranes were washed with wash buffer and incubated with streptavidin‐HRP for 30 min. Final detection was carried out by adding Chemi Reagent mix and chemiluminescence visualized using the Chemidoc™ MP (BioRad). Pixel density of each duplicate dot was collected and analyzed using ImageLab software. Positive reference spots were used to assess the test validity of the assay.

### Statistical analysis

Statistical analyses were performed using GraphPad Prism 7 software, version 7.0e. Data are shown as mean ± SD. Pairwise comparisons between patients and controls were performed using Student’s *t*-test for normally distributed variables, or Mann–Whitney *U* test for non-normally distributed variables and Chi squared test in categorical variables. More than two groups were compared using One-way ANOVA with Tukey´s post-hoc test or non-parametric analysis of variance Kruskal–Wallis test with a Dunn´s post-hoc multiple comparison test. Correlations between variables were analyzed with Pearson’s or Spearman´s coefficient depending on data distribution. Statistical significance was assumed if *P* ≤ 0.05.

### Ethics approval

The study was approved by the Clinical Research Ethic Committee from Hospital Universitari de Girona Dr. Josep Trueta (CEIm_COVID-Pneumo 2020.0099) in accordance with the Declaration of Helsinki.

### Consent to participate

All subjects gave written informed consent.

## Results

### Population characteristics

General characteristics of post-COVID-19 patients and healthy controls are shown in Table 1. Subjects were carefully selected and did not significantly differ in any of the characteristics analyzed, including age, sex, BMI, smoking history, and co-morbidities such as arterial hypertension (AHT), diabetes mellitus (DM) or dyslipidemia (DLP).

**Table 1:** General characteristics of COVID-19 patients and healthy controls

A							B					
Variables	CL, *n* = 31	3 months, post-COVID-19, *n* = 29	6 months post-COVID-19, *n* = 23	12 months post-COVID-19, *n* = 20	P-value (COVID-19 vs CL)	3 months post-COVID-19 w/o PE, *n* = 19	3 months post-COVID-19 with PE, *n* = 10	6 months post-COVID-19 w/o PE, *n* = 15	6 months post-COVID-19 with PE, *n* = 8	12 months post-COVID-19 w/o PE, *n* = 13	12 months post-COVID-19 with PE, *n* = 7	P-value (COVID-19 with PE vs w/o PE)
Age, years	58.6 ± 8.21	64.6 ± 13.8	64.2 ± 14.1	65.2 ± 13.8	ns	68.4 ± 10.6	57.3 ± 16.6	68.6 ± 9.01	55.9 ± 18.4^*^	68.8 ± 9.71	58.4 ± 18.2	P < 0.05
Male sex *n* (%)	20 (64.5%)	24 (82.8%)	19 (82.6%)	17 (85.0%)	ns	17 (89.5%)	7 (70.0%)	14 (93.3%)	5 (62.5%)	2 (15.4%)	1 (14.3%)	ns
BMI (Kg/m^2^)	26.7 ± 4.78	27.0 ± 3.17	26.7 ± 3.13	27.1 ± 4.7	ns	27.2 ± 3.03	26.5 ± 3.54	26.8 ± 3.23	26.5 ± 3.15	3.38 ± 0.94	5.39 ± 2.04	ns
Smokers (%)	1 (3.23%)	1 (3.45%)	1 (4.35%)	0 (0.00%)	ns	0 (0.00%)	1 (10.0%)	0 (0.00%)	1 (12.5%)	0 (0.00%)	0 (0.00%)	ns
No smokers (%)	19 (61.3%)	13 (44.8%)	10 (43.5%)	9 (45%)	ns	6 (31.6%)	7 (70.0%)	5 (33.3%)	5 (62.5%)	5 (38.5%)	4 (57.1%)	ns
Ex-smokers (%)	11 (35.5%)	15 (51.7%)	12 (52.2%)	11 (55%)	ns	13 (68.4%)	2 (20.0%)	10 (66.6%)	2 (25.0%)	8 (61.5%)	3 (42.9%)	ns
HTA (%)	7 (22.6%)	13 (44.8%)	11 (47.8%)	9 (45.0%)	ns	8 (42.1%)	6 (60.0%)	7 (46.7%)	4 (50.0%)	5 (38.5%)	4 (57.1%)	ns
DM (%)	0 (0.00%)	3 (10.3%)	2 (8.70%)	2 (10.0%)	ns	2 (10.5%)	1 (10.0%)	2 (13.3%)	0 (0.00%)	1 (7.69%)	1 (14.3%)	ns
DLP (%)	2 (6.45%)	5 (17.2%)	4 (17.4%)	3 (15.0%)	ns	3 (15.8%)	2 (20.0%)	(20.0%)	1 (12.5%)	1 (15.4%)	1 (14.3%)	ns

**A.** General characteristics of post-COVID-19 patients at different time points after overcoming COVID-19 (3, 6 and 12 months). Values expressed as mean ± SD. Ordinary one-way ANOVA followed by Tukey’s multiple comparisons test for parametric tests, Kruskal–Wallis test followed by Dunn’s multiple comparisons test for non-parametric tests and Chi squared test for categorical variables, *P* > 0.05. **B.** General characteristics of 3, 6 and 12-months post-COVID-19 patients, with or w/o pulmonary embolism (PE). Values expressed as mean ± SD. Unpaired *t* test for parametric tests, Mann–Whitney test for non-parametric tests and Chi squared test for categorical variables *P < 0.05

*CL* Healthy control, *PE* pulmonary embolism, *BMI* body mass index, *AHT* arterial hypertension, *DM* diabetes mellitus, *DLP* dyslipidemia

### Increased number of ECFCs in post-COVID-19 patients

Patients 3-months post-COVID-19 showed a significant increase in ECFC production compared to healthy subjects (82.8% *vs.* 48.4%, *P* < 0.01) which was maintained up to 6-months (87.0% *vs*. 48.4%, P < 0.01) and 12-months post-infection (85.0% *vs*. 48.4%, *P* < 0.01) (Fig. 1C; Table 2A). The average number of colonies in post-COVID-19 patients at 3 months (2.79 ± 2.41 *vs.* 1.23 ± 1.86, *P* < 0.05), 6 months (2.78 ± 2.00 *vs.* 1.23 ± 1.86, *P* < 0.05) and 12 months (2.90 ± 2.61 *vs.* 1.23 ± 1.86, *P* < 0.05) was also higher compared to healthy controls (Fig. 1D; Table 2). However, there was no difference regarding the appearance, number of ECFCs or the time needed for the colonies to emerge, between COVID-19 patients who suffered PE during admission than those that did not in any of the time-points studied (Table 2B).

**Table 2:** Cellular parameters collected from COVID-19 patients and healthy controls

A						B						
Variables	CL, *n* = 31	3 months post-COVID-19, *n* = 29	6 months post-COVID-19, *n* = 23	12 months post-COVID-19, *n* = 20	P-value (COVID-19 vs CL)	3 months post-COVID-19 w/o PE, *n* = 19	3 months post-COVID-19 with PE, *n* = 10	6 months post-COVID-19 w/o PE, *n* = 15	6 months post-COVID-19 with PE, *n* = 8	12 months post-COVID-19 w/o PE, *n* = 13	12 months post-COVID-19 with PE, *n* = 7	P-value (COVID-19 with PE vs w/o PE)
Appearance of ECFC colonies (%)	15 (48.4%)	24 (82.8%)**	20 (87.0%)**	17 (85.0%)**	P < 0.05	15 (79.0%)	9 (90.0%)	14 (93.3%)	6 (75.0%)	11 (84.6%)	6 (85.7%)	ns
Number of ECFC colonies	1.23 ± 1.86	2.79 ± 2.41*	2.78 ± 2.00*	2.90 ± 2.61*	P < 0.05	2.47 ± 2.17	3.40 ± 2.84	2.73 ± 1.62	2.88 ± 2.70	2.62 ± 2.29	3.43 ± 2.26	ns
Time for ECFC to appear (days)	14.3 ± 4.76	11.6 ± 4.73	10.8 ± 3.23	12.1 ± 4.10	ns	11.7 ± 3.81	11.4 ± 6.23	11.6 ± 3.25	8.67 ± 2.16	12.1 ± 4.89	12.0 ± 2.45	ns

*CL* healthy control, *PE* Pulmonary embolism, *ECFC* endothelial colony-forming cells

A. Cellular parameters of post-COVID-19 patients at different time points after overcoming COVID-19 (3, 6 and 12 months). Values expressed as mean ± SD. Ordinary one-way ANOVA followed by Tukey´s multiple comparisons test for parametric tests, Kruskal–Wallis test followed by Dunn’s multiple comparisons test for non-parametric tests and Chi squared test for categorical variables, *P < 0.05, **P < 0.01

B. Cellular parameters of 3,6 and 12-months post-COVID-19 patients, with or w/o pulmonary embolism (PE). Values expressed as mean ± SD. Unpaired *t* test for parametric tests, Mann–Whitney test for non-parametric tests and Chi squared test for categorical variables, *P* > 0.05

### Higher levels of troponin, NT-proBNP and ferritin in post-COVID-19 patients

Clinical parameters collected from post-COVID-19 patients and healthy subjects are shown in Table 3. Troponin levels were significantly higher in 3-months post-COVID-19 patients compared to healthy controls (19.6 ± 13.6 *vs.* 6.46 ± 3.61, *P* < 0.0001) and remained elevated at 6-months (16.2 ± 14.3 *vs.* 6,46 ± 3,61, *P* < 0.05) and 12-months after SARS-CoV-2 infection (14.8 ± 11.4 *vs.* 6.46 ± 3.61, *P* < 0.01) (Fig. 1E; Table 3). NT-proBNP levels were also significantly higher at 3-months post-COVID-19 compared to healthy controls (415 ± 640.3 vs. 127 ± 65.0, P < 0.01) and remained elevated up to 6-months (442 ± 670 vs. 127 ± 65.0, *P* < 0.05) and 12 months after infection (222 ± 176 *vs.* 127 ± 65.0, *P* < 0.05) (Fig. 1F; Table 3).

**Table 3:** Clinical parameters collected from COVID-19 patients and healthy controls

Variables	CL, *n* = 31	3 months post-COVID-19, *n* = 29	6 months, post-COVID-19, *n* = 23	12 months post-COVID-19, *n* = 20	*P*-value, (COVID-19 vs CL)
FVC (%)	93.8 ± 16.6	98.7 ± 16.6	98.8 ± 20.4	104 ± 21.3	ns
FEV_1_ (%)	98.4 ± 17.9	93.2 ± 28.1	101 ± 20.3	102 ± 25.7	ns
FEV_1_/FVC (%)	79.7 ± 4.79	94.8 ± 23.3**	64.0 ± 52.5	96.9 ± 12.1	< 0.05
TLC (L)	ND	109 ± 27.5	96.1 ± 9.45	96.8 ± 15.3	ns
RV (L)	ND	108 ± 25.3	94.8 ± 19.1	89.1 ± 32.5	ns
DLCO (%)	ND	73.7 ± 17.2	73.9 ± 8.74	67.3 ± 11.9	ns
6MWT (m)	ND	360 ± 73.5	372 ± 127	366 ± 118	ns
Hb (g/dL)	14.4 ± 1.61	13.8 ± 1.74	14.0 ± 1.74	13.9 ± 1.84	ns
HTC (%)	43.2 ± 4.00	42.7 ± 4.81	43.1 ± 4.40	43.0 ± 4.64	ns
Lym (K/mcL)	1.94 ± 0.54	2.35 ± 0.92	1.91 ± 0.65	2.06 ± 0.59	ns
LDH (mg/dL)	180 ± 18.4	192 ± 32.7	198 ± 37.3	198 ± 30.0	ns
MF (ng/mL)	108 ± 93.9	143 ± 116	144 ± 117	159 ± 128	ns
Iron (II,III) (µg/dL)	87.3 ± 30.9	72.8 ± 28.2*	83.0 ± 24.1	89.7 ± 38.1	< 0.05
Transferrin (mg/dL)	251 ± 41.2	252 ± 41.2	271 ± 55.4	254 ± 76.7	ns
Transferrin saturation (%)	25.1 ± 9.52	20.6 ± 7.81*	22.5 ± 8.39	25.4 ± 12.7	< 0.05
CRP (mg/dL)	0.11 ± 0.06	0.32 ± 0.59	0.29 ± 0.25	0.50 ± 1.05	ns
Troponin T(ng/L)	6.46 ± 3.61	19.6 ± 13.6****	16.2 ± 14.3*	14.8 ± 11.4**	< 0.05
NT-proBNP (pg/mL)	127 ± 65.0	415 ± 640.3**	442 ± 670*	222 ± 176*	< 0.05
Positive DD (%)	1 (9.09%)	5 (19.2%)	5 (23.8%)	3 (15.0%)	ns
FIB (mg/dL)	410 ± 61.1	409 ± 84.2	450 ± 78.9	448 ± 90.9	ns

Clinical characteristics of 3, 6 and 12-months post-COVID-19 patients and healthy controls. Values expressed as mean ± SD

*CL* Healthy control, *FVC* forced vital capacity, *FEV* forced expiratory volume, *TLC* total lung capacity, *RV* residual volume, *DLCO* carbon monoxide diffusing capacity, *6MWT* Six minute walk test, *Hb* hemoglobin, *HTC* hematocrit, *Lym* lymphocytes, *LDH* lactate Dehydrogenase, *MF* maximum ferritin, *CRP* C reactive protein, *NT-proBNP* N-terminal-pro hormone B-type natriuretic peptide, *DD* Dimer-D, *FIB* fibrinogen

Ordinary one-way ANOVA followed by Tukey’s multiple comparisons test for parametric tests and Kruskal–Wallis test followed by Dunn’s multiple comparisons test for non-parametric tests, *P < 0.05, **P < 0.01, ****P < 0.0001. Reference values: Troponin, 0–14 pg/mL; Lymphocytes, 1–4,5 (K/mcL); LDH, 135–225 (mg/dL); Ferritin, 30–400 (ng/mL)

Additionally, the subgroup analysis performed between PE and non-PE post-COVID-19 patients (Table 4) showed that post-COVID-19 patients without PE presented higher troponin levels at all the time-points analyzed, compared to post-COVID-19 who suffered PE. This increase reached significance at 6-months post-infection (20.0 ± 15.1 *vs.* 8.69 ± 9.12, *P* < 0.05). Moreover, non-PE patients also showed significant elevation of NT-proBNP levels at 3 (581 ± 791 *vs.* 166 ± 109, *P* < 0.05) and 6-months (656 ± 805 *vs.* 121 ± 64.3, *P* < 0.05) post-infection. Interestingly, troponin levels correlated positively with NT-proBNP levels in 6-months post-COVID-19 patients (*r* = 0.65, *P* < 0.01) (Supplementary Table S2).

**Table 4:** Clinical parameters collected from COVID-19 patients

Variables	3 months, post-COVID-19 w/o PE, *n* = 19	3 months, post-COVID-19 with PE, *n* = 10	6 months, post-COVID-19 w/o PE, *n* = 15	6 months, post-COVID-19 with PE, *n* = 8	12 months, post-COVID-19 w/o PE, *n* = 13	12 months, post-COVID-19 with PE, *n* = 7	*P*-value, (COVID-19 with PE, vs w/o PE)
FVC (%)	97.8 ± 18.0	101 ± 13.8	97.8 ± 23.9	102 ± 4.95	107 ± 27.5	100 ± 5.57	ns
FEV_1_ (%)	90.6 ± 30.8	98.9 ± 21.6	99.2 ± 23.6	107 ± 4.24	102 ± 33.6	101 ± 7.23	ns
FEV_1_/FVC (%)	93.5 ± 26.8	97.5 ± 14.4	68.7 ± 52.8	50.1 ± 69.2	94.5 ± 101	101 ± 8.49	ns
TLC (L)	111 ± 32.8	105 ± 15.1	96.3 ± 10.6	95.5 ± 7.78	101 ± 18.7	90.3 ± 4.51	ns
RV (L)	105 ± 20.4	114 ± 32.8	101 ± 17.1	75.5 ± 10.6	99.2 ± 38.7	72.3 ± 6.03	ns
DLCO (%)	73.3 ± 19.8	74.3 ± 11.4	76.5 ± 8.60	66.0 ± 0.00	69.2 ± 14.3	64.0 ± 7.81	ns
6MWT (m)	342 ± 80.4	397 ± 39.3	316 ± 117	483 ± 0.00	334 ± 121	420 ± 111	ns
Hb (g/dL)	13.7 ± 1.71	14.2 ± 1.92	14.1 ± 1.60	13.7 ± 2.12	14.0 ± 1.83	13.8 ± 2.00	ns
HTC (%)	42.3 ± 5.01	43.5 ± 4.59	43.4 ± 4.36	42.4 ± 4.76	43.4 ± 4.96	42.2 ± 4.25	ns
Lym (K/mcL)	2.4 ± 0.98	2.17 ± 0.79	1.85 ± 0.63	2.01 ± 0.74	1.97 ± 0.66	2.24 ± 0.45	ns
LDH (mg/dL)	188 ± 33.4	203 ± 31.3	195 ± 37.2	205 ± 39.6	192 ± 33.9	208 ± 18.6	ns
MF (ng/mL)	145 ± 103	136 ± 157	180 ± 123	72.7 ± 63.0^*^	168 ± 120	143 ± 151	< 0.05
Iron (II,III) (µg/dL)	67.4 ± 24.9	78.1 ± 29.9	81.0 ± 27.4	85.3 ± 21.5	93.2 ± 48.6	88.1 ± 33.5	ns
Transferrin (mg/dL)	255 ± 57.4	250 ± 47.6	284 ± 48.4	260 ± 56.0	270 ± 60.2	245 ± 81.0	ns
Transferrin saturation (%)	19.8 ± 8.56	22.4 ± 8.65	20.8 ± 8.67	23.9 ± 7.79	26.3 ± 16.2	25.1 ± 11.21	ns
CRP (mg/dL)	0.41 ± 0.68	0.10 ± 0.06	0.31 ± 0.27	0.24 ± 0.20	0.32 ± 0.32	0.85 ± 1.74	ns
Troponin (ng/L)	21.8 ± 13.9	13.5 ± 11.8	20.0 ± 15.1	8.69 ± 9.12^*^	17.8 ± 13.1	9.69 ± 5.11	< 0.05
NT-proBNP (pg/mL)	581 ± 791	166 ± 109*	656 ± 805	121 ± 64.3**	278 ± 210	144 ± 40.5	< 0.05
Positive DD (%)	5 (29.4%)	0 (0.00%)	3 (21.4%)	2 (28.6%)	2 (15.4%)	1 (14.3%)	ns
FIB (mg/dL)	418 ± 80.4	385 ± 96.8	456 ± 84.4	440 ± 72.3	440 ± 79.2	464 ± 115	ns

Clinical characteristics of 3, 6 and 12-months post-COVID-19 patients with or w/o pulmonary embolism (PE)

*PE* pulmonary embolism, *FVC* forced vital capacity, *FEV* forced expiratory volume, *TLC* total lung capacity, *RV* residual volume, *DLCO* carbon monoxide diffusing capacity, *6MWT* Six minute walk test, *Hb* hemoglobin, *HTC* hematocrit, *Lym* lymphocytes, *LDH* lactate dehydrogenase, *MF* maximum ferritin, *CRP* C reactive protein, *NT-proBNP* N-terminal-pro hormone B-type natriuretic peptide, *DD* Dimer-D, *FIB* Fibrinogen

Values expressed as mean ± SD. Unpaired *t* test for parametric tests, Mann–Whitney test for non-parametric tests and Chi squared test for categorical variables, *P < 0.05

Iron (II,III) levels (87.3 ± 30.9 *vs.* 72.8 ± 28.2, *P* < 0.05) and transferrin saturation (25.1 ± 9.52 *vs.* 20.6 ± 7.81, *P* < 0.05) were significantly decreased 3-months post-COVID-19 patients compared to healthy controls (Table 3). However, no correlations were found between iron levels or transferrin saturation and ferritin, hemoglobin (Hb) or hematocrit (HTC) levels. Interestingly, although no differences were found in ferritin levels between post-COVID-19 patients and healthy subjects, ferritin levels were also increased in post-COVID-19 subjects without PE compared to those patients who suffered PE, being also statistically significant at 6-months post-COVID-19 (180 ± 123 vs*.* 72.7 ± 63.0, *P* < 0.05) (Table 4).

### Correlations between ECFC levels and clinical parameters

**Fig. 2:**
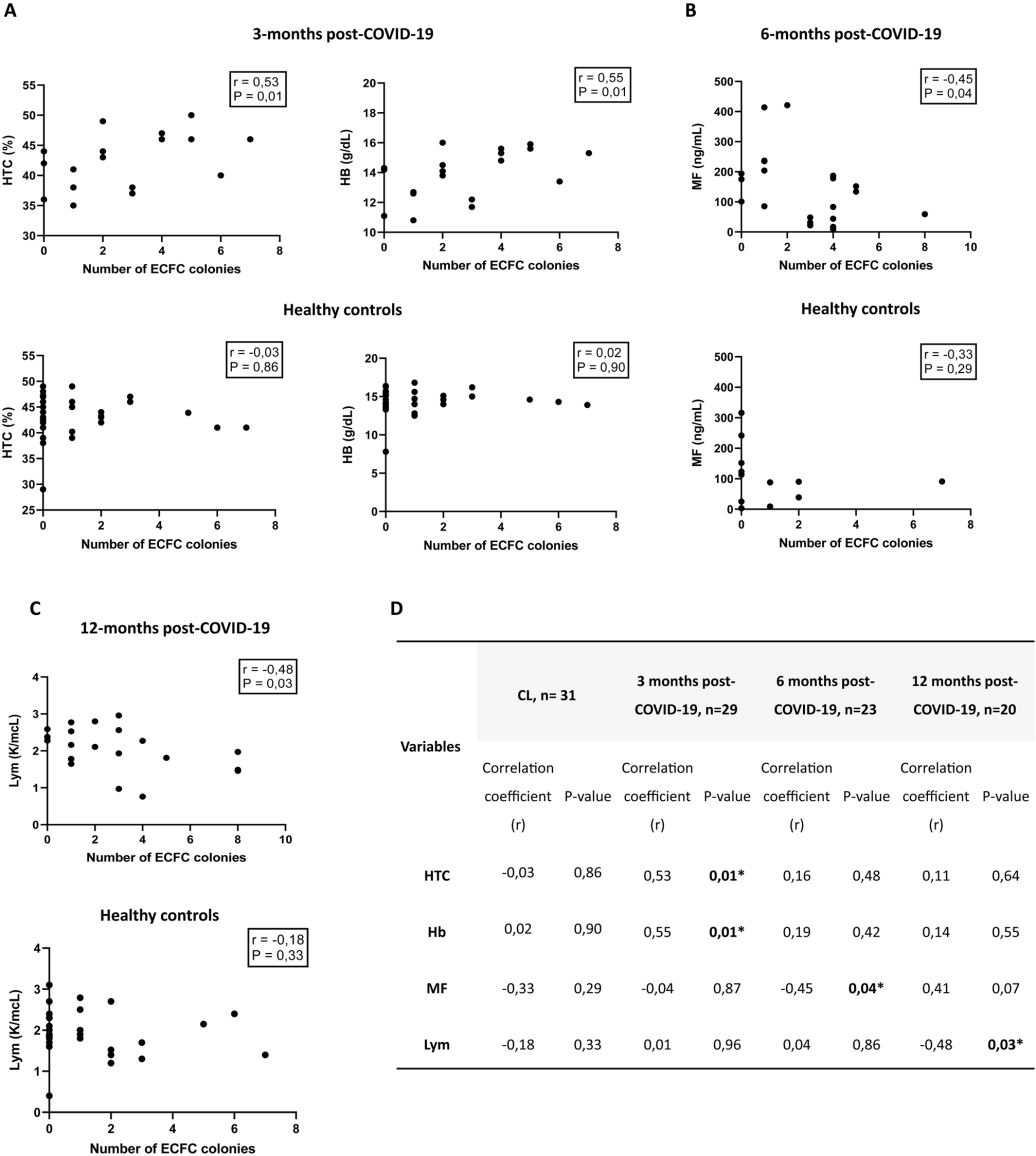
Relationship between number of ECFC colonies and clinical characteristics in COVID-19 patients and healthy controls. **A** Correlation between HTC and Hb and number of ECFC colonies at 3 months post-COVID-19 and healthy controls, Spearman rank correlation, *P < 0.05. **B** Correlation between MF levels and number of ECFC colonies at 6 months post-COVID-19 and healthy controls, Spearman rank correlation, *P < 0.05. **C** Correlation between lymphocytes levels and number of ECFC colonies at 12 months post-COVID-19 and healthy controls, Spearman rank correlation, *P < 0.05. **D** Correlation coefficients (r) and p-values for the relationship between the number of ECFC colonies and clinical characteristics in COVID-19 patients at 3, 6 and 12-months after infection and healthy controls. Spearman rank correlation, *P < 0.05**.** *CL* Healthy controls, ECFC endothelial colony-forming cells, *HTC* hematocrit, *Hb* hemoglobin, *MF* maximum ferritin

As shown in Fig. 2A, patients who had higher numbers of ECFC colonies presented higher levels of Hb and HTC at 3-months post-infection. Conversely, patients who showed lower numbers of ECFC colonies had higher levels of ferritin (*r* = – 0.45, *P* = 0.04) at 6 months after overcoming COVID-19 (Fig. 2B). Number of ECFC colonies also correlated negatively with lymphocyte levels (*r* = – 0.48, *P* = 0.03) at 12-months post-COVID-19 patients (Fig. 2C). No correlation was found between ECFC colonies and troponin or NT-proBNP levels.

### Reduced angiogenic profile in post-COVID-19 patients

**Fig. 3:**
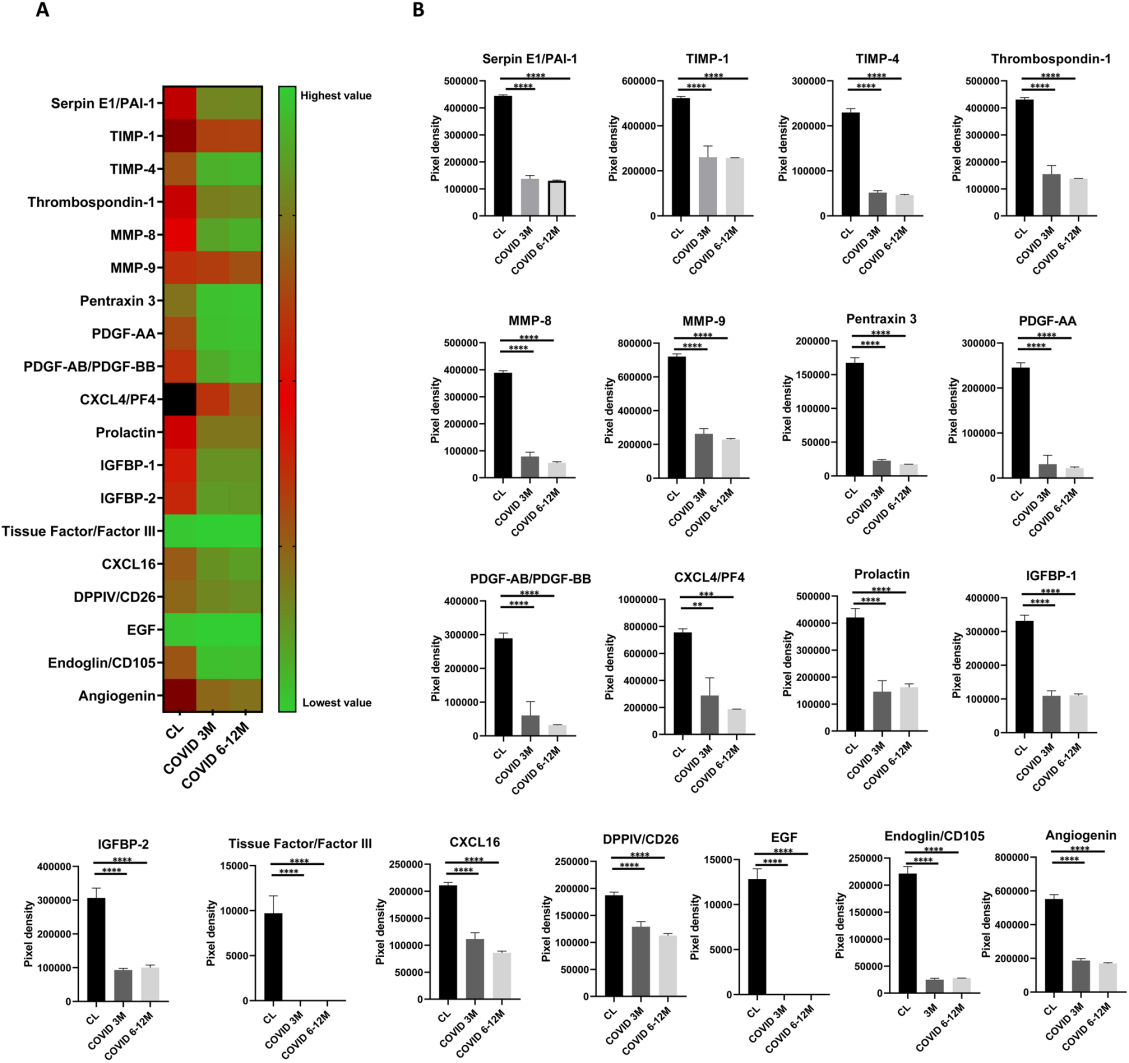
**A** Heat map of mean values of the angiogenesis-related proteins significantly expressed between healthy controls and post-COVID-19 patients at short term (3-months) and long term (6–12 months). Green represents low expression, red moderate expression and black high expressed factors. **B** Pixel density of the angiogenesis-related proteins significantly expressed between healthy controls and post-COVID-19 patient. Ordinary one-way ANOVA followed by Tukey´s multiple comparisons test, ****P < 0.001. *PAI-1* Plasminogen activator inhibitor 1, *TIMP-1* tissue inhibitor of metalloproteinase 1, *TIMP-2* Tissue inhibitor of metalloproteinase 2, *MMP-8* matrix metalloproteinase-8, *MMP-9* matrix metalloproteinase-9, *PDGF-AA* platelet-derived growth factor subunit A, *PDGF-BB* platelet-derived growth factor subunit B, *CXCL4* platelet factor 4, *PF4* platelet factor 4, *IBP-1* Insulin-like growth factor-binding protein 1, *IBP-2* insulin-like growth factor-binding protein 2, *CXCL16* platelet factor 16, *DPPIV* dipeptidyl peptidase IV, *CD26* Cluster of differentiation 26, *EGF* epidermal growth factor

**Fig. 4:**
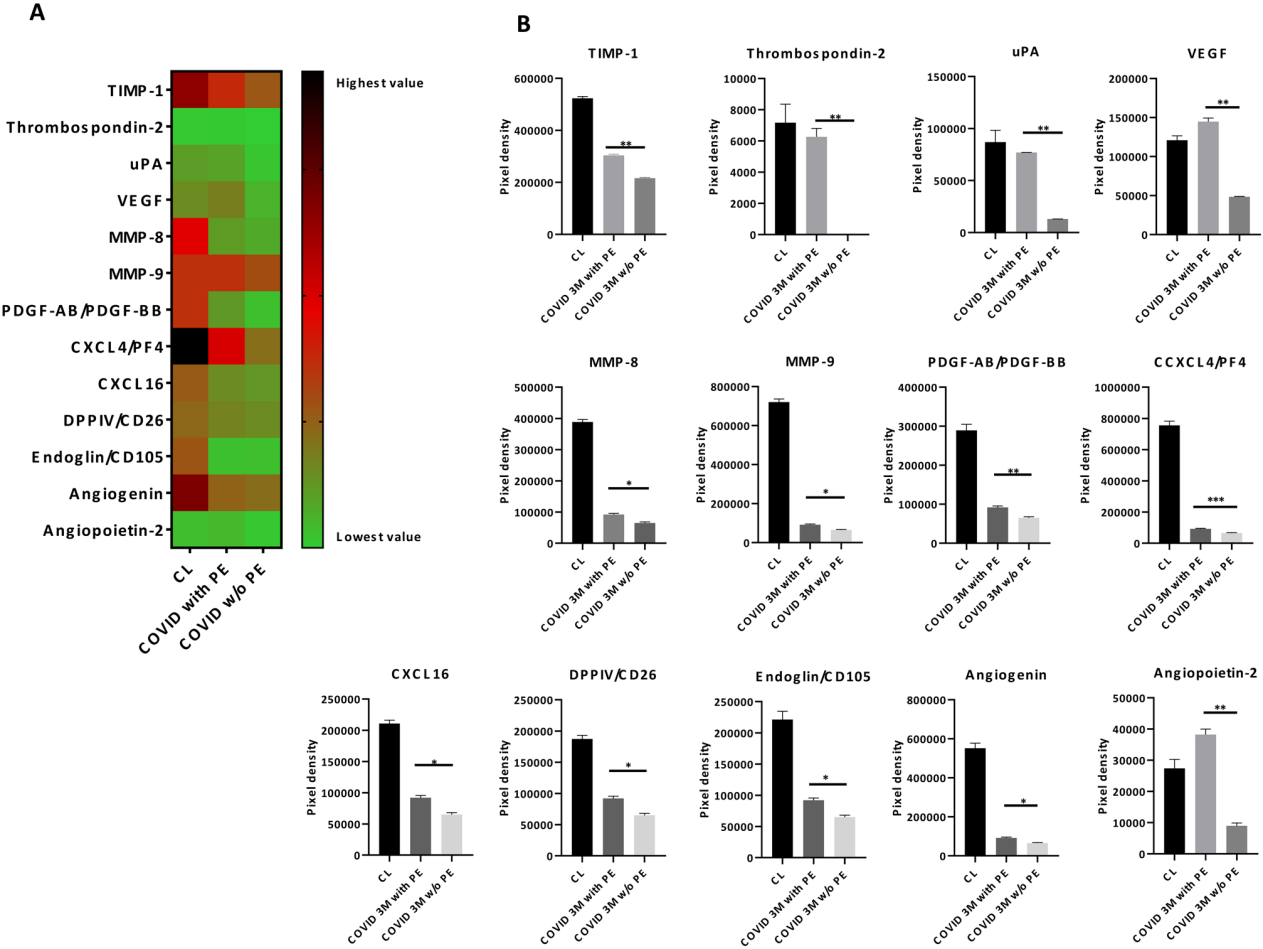
**A** Heat map of mean values of the angiogenesis-related proteins significantly expressed between 3-months post-COVID-19 patients with or w/o pulmonary embolism (PE) and healthy controls. Green represents low expression, red moderate expression, and black high expressed factors. **B** Pixel density of the angiogenesis-related proteins significantly expressed between 3-months post-COVID-19 patients with or w/o pulmonary embolism (PE) and healthy controls. Unpaired *t* test, *P < 0.05, **P < 0.01, ***P < 0.001. *TIMP-1* tissue inhibitor of metalloproteinase 1, *uPA* urokinase plasminogen activator, *VEGF* vascular endothelial growth factor, *MMP-8* matrix metalloproteinase-8, *MMP-9* matrix metalloproteinase-9, *PDGF-AA* platelet-derived growth factor subunit A, *PDGF-BB* platelet-derived growth factor subunit B, *CXCL4* platelet factor 4, *PF4* platelet factor 4, *CXCL16* platelet factor 16, *DPPIV* dipeptidyl peptidase IV (*DPPIV*), *CD26* cluster of differentiation 26

19 of the 55 angiogenesis-related proteins analyzed such as TIMP-1/4, MMP-8/9, PDGF-AA/AB-BB, CXCL4/16, IGFBP-1/2, Tissue Factor/Factor III, Endoglin or Angiogenin were significantly downregulated at 3-months post-COVID-19 patients compared to healthy controls and this decrease remained significantly downregulated at 6 and 12-months post-infection (Fig. 3A, B). Additionally, 13 of 55 angiogenic proteins including TIMP-1, uPA, VEGF, MMP-8/9, CXCL4/16, Endoglin, Angiogenin or Angiopoietin-2 were uniquely downregulated in 3-months post-COVID-19 patients without PE compared to those who suffered a PE (Fig. 4A, B).

### Long-COVID and sequela incidence

Long-COVID symptoms were described in 67,9%, 52,2% and 42,1% of 3, 6 and 12-months post-COVID-19 patients, respectively (Supplementary Table S3A). As shown in Supplementary Table S3B, fatigue (25.0%), dyspnea (16.7%) or chest pain (14.3%) at 3-months, dyspnea (66.7%), fatigue (17.4%) or anxiety (8.70%) at 6 months, and dyspnea (42.9%), joint pain (10.5%) or loss of memory (10.5%) at 12 months were the most common symptoms. The presence of long-COVID pathology was associated with higher ferritin levels at 3-months post-COVID-19 and with lower levels of hemoglobin (*r* = – 0.43, *P* = 0.049) at 6-months. Interestingly, long-COVID significantly correlated with the female gender (*r* = 0.40, *P* = 0.036). However, no correlation was found between long-COVID pathology and the appearance, number of ECFC colonies or any other parameter measured (Supplementary Table S4).

## Discussion

Patients 3 months after SARS-CoV-2 infection, presented an endothelial progenitor cell imbalance with an abnormal increase of circulating ECFCs when compared to healthy subjects [21]. Noteworthy, this increase in circulating ECFCs stayed in post-COVID-19 patients up to 6 and 12 months indicating an abnormal ECFC mobilization in response to a persistent vascular damage. The long-term effects of this vascular damage are unknown and deserve long-term follow up studies.

Our data show that COVID-19 not only triggers a prompt endothelial dysfunction but also stimulates the mobilization and recruitment of endothelial progenitor cells as a response to a vascular damage and promote angiogenesis and vascular repair. In line with these results, Mancuso et al. [20]*.* recently reported that endothelial progenitor cells, identified by flow cytometry as CD45^−^CD34^+^CD31^+^CD146^−^, were significantly increased in COVID‐19 patients compared with healthy controls. Additionally, Alvarado-Moreno et al. [31]*.* found an increase of ECFCs in COVID-19 recovered patients as compared with controls. Conversely, the ability of these isolated ECFCs to form new blood vessels was significantly reduced [31].

Our results showed no significant difference in the appearance or number of ECFC colonies generated between COVID-19 patients who suffered PE during admission and those that did not. Endothelial progenitor cells have been shown to participate in thrombus resolution, restoring the damaged endothelium and promoting neovascularization [32]. Consequently, a higher ECFC mobilization could be anticipated in patients after a PE. Unexpectedly, we did not observe greater ECFC numbers in PE post-COVID-19 patients compared to non-PE patients in any of the time points analyzed. This result reflects that the increase of circulating ECFC in COVID-19 is related to the significant endothelial injury produced by the infection itself rather to the development of PE during hospital admission.

Troponin levels also remained elevated in post-COVID-19 patients up to 12 months after the infection. Troponin not only is a biomarker of cardiac injury but also of systemic inflammation. In our series, post-COVID-19 patients showed higher troponin levels at 3-months post-infection compared to healthy controls, which was maintained up to 6 and 12 months. Several studies have reported an increase in troponin levels and the presence of myocardial injury during the acute phase of COVID-19 [33, 34] and after clinical resolution [27, 35]. Puntmann et al. [27]*.* revealed that 78% of patients recently recovered from COVID-19 had cardiac involvement and 60% of these subjects showed ongoing myocardial inflammation, independent of preexisting conditions, detected by cardiac magnetic resonance imaging (MRI). Additionally, troponin can be increased as a result of systemic inflammation. Søyseth et al*.* [36] showed that COPD patients exhibited an increase in troponin levels caused by the systemic inflammation that occurs during exacerbation and pneumonia.

Higher troponin levels were observed in non-PE post-COVID-19 patients compared to PE patients. PE is clinically characterized by higher troponin levels, but the relationship between COVID-19 disease and the presence of a PE is still under debate. Recent studies point out to the existence of differential clinical characteristics between the development of PE in COVID-19 patients compared to patients with PE without COVID-19. Miró et al*.* [37] reported that COVID-19 patients with a PE exhibited a more discrete rise in D-dimers compared to patients with PE without COVID-19 and that the thrombi produced in COVID-19 patients affected mainly smaller pulmonary arteries. Moreover, leg swelling, pain and risk factors for PE were significantly less in COVID-19 than in non-COVID-19 patients with PE, suggesting that artery thrombosis could have been developed in situ within the lungs, stimulated by a high inflammatory environment [37]. While these results might seem counterintuitive, higher levels of inflammatory markers in non-PE patients secondary to the SARS-CoV-2 infection might have induced a significant rise of troponin levels. These findings seem to indicate that the massive inflammation response in the peripheral lung vessels produced by SARS-CoV-2 could hide the effect of the PE suffered.

NT-proBNP levels also remained elevated in post-COVID-19 patients up to 12-months after the infection. As troponin, NT-proBNP not only is used as a biomarker for impaired cardiac function and heart failure, but also is an indicator of inflammation [38, 39]. Elevated NT-proBNP levels have been previously reported in COVID-19 patients and in post-COVID-19 patients. Gul et al*.* [40] described that NT-ProBNP levels were significantly higher in patients who had recovered from COVID-19 compared to the control group, suggesting the presence of heart damage and stress. Our data shows that non-PE post-COVID-19 patients had higher NT-proBNP levels when compared to PE patients. Furthermore, our study revealed a correlation between NT-proBNP and troponin. These findings collectively seem to indicate that non-PE patients experienced more pronounced inflammatory conditions.

In our cohort, higher ferritin levels, and lower levels of iron and transferrin saturation were also observed in post-COVID-19 patients compared to healthy controls. Ferritin, the main intracellular iron storage protein, is considered an acute phase reactant which is increased in acute inflammatory conditions, including severe infections [41]. Higher ferritin levels have been observed in COVID-19 patients and in post-COVID-19 patients after discharge [42, 43]. Recent data reported that iron metabolism is affected by SARS-CoV-2 infection, producing an increase in cytosolic ferritin that stores the iron to prevent iron-mediated free radical damage, a process called ferroptosis [43, 44]. The body tries to self-protect against this damage, increases the levels of ferritin and decreases the availability of iron [45]. Moreover, high ferritin levels and low transferrin saturation has been reported to be associated with an increased risk of cardiovascular disease [46]. Ferritin levels also may be upregulated secondary to compensatory activation of heme oxygenase I (HO-1), an enzyme responsible for the oxidative cleavage of heme groups. Hemoglobin, when is oxidized, transfer heme group to endothelium, which upregulates heme oxygenase-1 and ferritin as a defense mechanism [47, 48]. Moreover, this protein not only is an indicator of inflammation but also could be a direct indicator of cellular damage [43]. Similarly, non-PE post-COVID-19 patients showed higher ferritin levels compared to those who suffered a PE.

Post-COVID-19 patients with lower numbers of ECFC colonies showed higher levels of ferritin, and lower levels of hemoglobin suggesting a deficient ECFC mobilization response to the endothelial damage. We have previously described that patients with higher numbers of ECFC colonies presented higher levels of hemoglobin, a protective response to compensate the hypoxic conditions suffered to quickly restore the damaged endothelium [21]. Additionally, in this study we found that recovered patients with lower numbers of ECFC colonies had higher levels of ferritin, suggesting that those patients presented a higher inflammatory response, possibly as a result of an insufficient ECFC mobilization that could act as a protective mechanism. Therefore, these results could indicate for the first time that the generation of higher number of ECFC colonies after the infection could be related to a better prognosis in COVID-19 patients.

Furthermore, our findings reveal that post-COVID-19 patients show a significant downregulation of angiogenesis-related proteins at 3 months that persists up to 12-months post-infection. Increased evidence of altered endothelial and angiogenic markers have been observed in several reports. In COVID-19 patients during admission, increased levels of circulating pro-angiogenic factors such as VEGF-A, PDGF-AA, and PDGF-AB/BB were found in patients with COVID-19 compared to healthy controls, contributing to the vascular remodeling processes and the formation of new blood vessel in COVID-19 [49]. Beltrán-Camacho et al. [50]*.* demonstrated that the serum from COVID-19 asymptomatic patients upregulate proteins related to endothelial dysfunction in circulating angiogenic cells (CACs). Moreover, Sibila et al. [18] observed elevated levels of endothelial markers in COVID-19 patients with reduced DL_CO_ levels 6-months after hospital discharge, suggesting the presence of persistent lung damage. Willems et al*.* [51] found elevated circulating inflammatory cytokines (IL-6), sustained coagulation (FVII:AT, TAT) and endothelial cell activation (vWF) 18 months after COVID-19 infection. However, they did not observe an increased macrovascular dysfunction. All these markers can reflect an endothelial activation after infection. Nevertheless, our results showed a clear dysregulation of the angiogenic function in post-COVID-19 patients, which is in accordance with the reduced ability of ECFCs to form new blood vessels in post-COVID-19 patients reported by Alvarado-Moreno et al*.* [31]. It has been suggested that ECFCs from recovered COVID-19 patients showed an abnormal function, resulting in the absence or reduction of endothelial regeneration as well as their ability to properly respond to stimulation with plasma from recovered COVID-19 patients [31]. These results suggest that the reduced angiogenic capacity observed in these patients could reflect a greater endothelial lesion. Similarly, endothelial dysfunction has been shown to impair angiogenic processes [52]. In line of our results, we also showed a loss of angiogenic capacity as a response to an endothelial damage. The increased endothelial damage is counteracted by an increase of a ECFC mobilization into the systemic circulation to rapidly regenerate the damaged endothelium.

Additionally, non-PE subjects also showed a significant downregulation of angiogenesis-related proteins compared to PE post-COVID-19 patients, indicating higher vascular damage. Thrombosis related-proteins, such as urokinase-type plasminogen activator (uPA), were significantly increased in PE subjects compared to non-PE post-COVID-19 patients. uPA is a protein involved in clot resolution by catalyzing the conversion of plasminogen to plasmin, and promote cell migration after endothelial cell injury [53, 54]. As pulmonary embolism has been reported to commonly resolve within 6 months [55] higher levels of uPA and fibrinolytic capacity [55] in PE patients is not unexpected. Lang et al*.* [54] found an increase in uPA expression during vascular remodeling in pulmonary artery specimens from patients suffering PE. Conversely, angiogenic compounds such as VEGF or angiopoietin-2 were significantly downregulated in non-PE post-COVID-19 subjects compared to PE. Bontekoe et al*.* [56] reported an upregulation of VEGF, a potent angiogenic cytokine, in plasma samples from PE subjects compared to healthy subjects. Moreover, angiopoietin-2 is expressed in tissues undergoing vascular remodeling, and both hypoxia and VEGF upregulate angiopoietin-2 in endothelial cells [57]. These findings together with the increase in troponin and inflammatory biomarkers could indicate a greater vascular damage in non-PE post-COVID-19 patients compared to those with PE. The clinical relevance, outcomes, and risks between these two subgroups of COVID-19 patients are currently unknown and future long-term studies are needed.

In agreement with current data, our results show that 80% of COVID-19 patients developed at least one long term-symptom, being the most frequent symptoms fatigue, dyspnea or attention disorders [58]. We also found that the presence of this syndrome was associated with higher ferritin levels and lower hemoglobin levels. As ferritin is an indicator of inflammation and cellular damage, it can suggest that long-COVID patients developed these symptoms due to the persistent inflammatory response. Therefore, persistent inflammatory response and reduced levels of hemoglobin could explain some of the long-COVID symptoms. These biomarkers could potentially be employed for the early identification of long-term sequelae, defined as a condition or complication resulting from a previous disease and lasting over time.

Interestingly, long-COVID pathology was also related to female gender. To date, several studies found that females were more likely to develop long-term symptoms, being a risk factor for persistent symptoms [59, 60]. However, it is not clear why women were more likely to present long-COVID symptoms.

## Conclusion

**Fig. 5:**
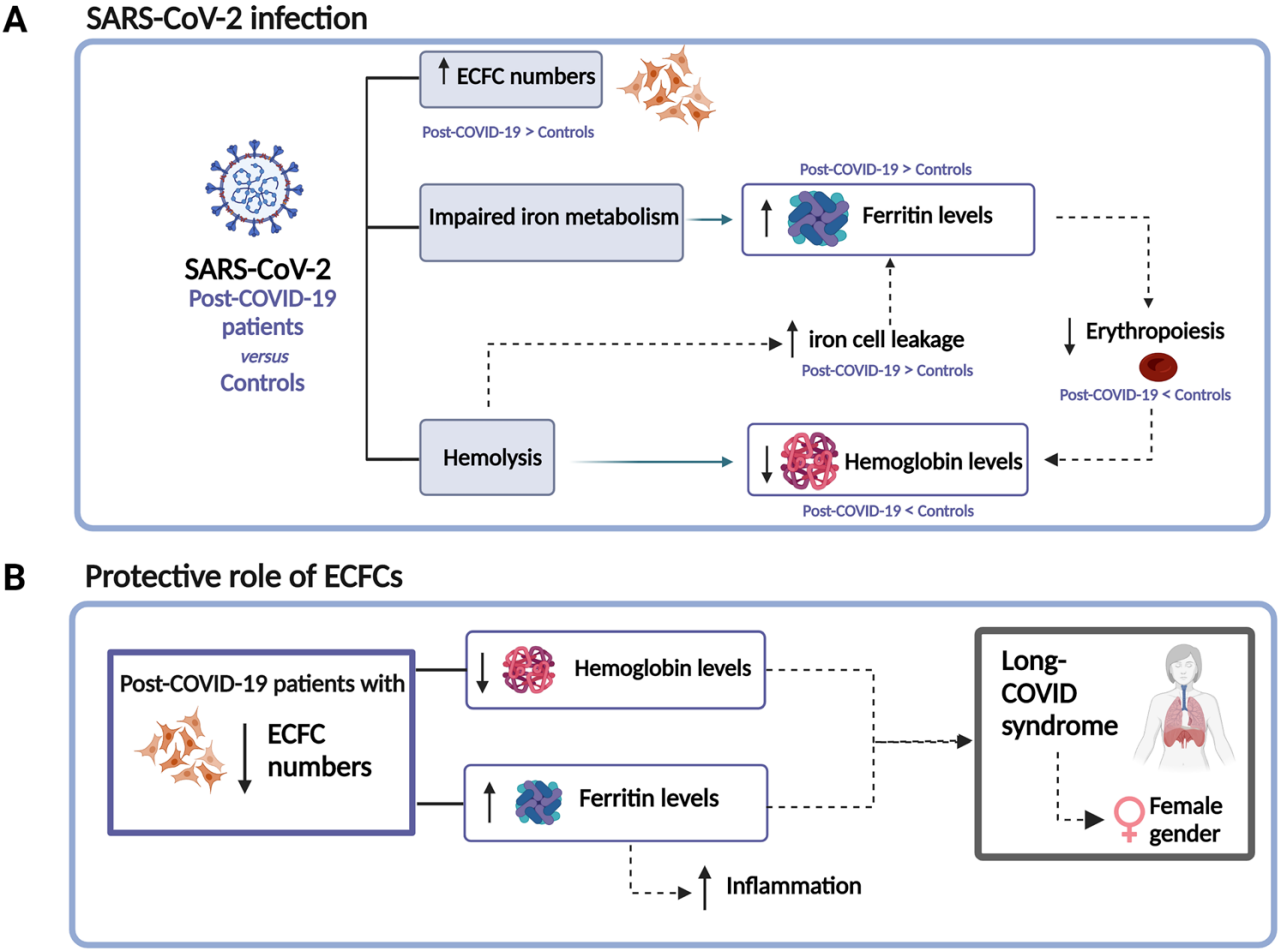
**A** SARS-CoV-2 infection produces an abnormal increase of circulating ECFCs and impairs iron metabolism increasing the levels of ferritin and decreasing erythropoiesis, resulting in a decrease in hemoglobin levels. Additionally, SARS-CoV-2 causes digestion of hemoproteins, decreasing the levels of hemoglobin and resulting in iron cell leakage, which increase ferritin levels. **B** Post-COVID-19 patients with lower numbers of ECFC had lower levels of hemoglobin and higher levels of ferritin, suggesting that those patients presented a higher inflammatory response, possibly as a result of an insufficient ECFC mobilization that could act as a protective mechanism. Long-COVID syndrome was associated with higher ferritin levels, lower hemoglobin levels and with female gender. *ECFC* endothelial colony-forming cells. Created with BioRender.com

Overall, our findings confirm that the presence of the vascular sequelae found in 3-months post-COVID-19 patients persists up to 6 and 12-months post-infection with an imbalance in ECFC number and a significant downregulation of angiogenesis-related proteins. These findings highlight the importance of a patient follow-up measuring the levels of ECFCs, troponin, NT-proBNP and ferritin among the rest of biomarkers (Fig. 5).

### Study limitations

This study has some limitations. As the plasma samples used for the angiogenic assay were a pool of subjects, we cannot correlate the levels of expression of the different angiogenic factors and the number or appearance of ECFC colonies of each subject. Nevertheless, our results show a clear profile of post-COVID-19 pathology. An additional limitation of our study is that although we suggest that the increase of ECFC is associated with an endothelial dysfunction, we were unable to definitively confirm it, primarily due to the absence of an assessment for pre-existing endothelial dysfunction in the subjects. Moreover, it is important to note that the sample size of our study is relatively small and increasing it would provide more precise results and enhance the significance level of our findings.

### Supplementary Information

Below is the link to the electronic supplementary material.

Supplementary file1 (DOCX 615 KB)

## Data Availability

All data relevant to the study are included in the article or uploaded as supplementary information.
