# Supplementary material for: Endothelial dysfunction and cardiovascular risk in post-COVID-19 patients after 6- and 12-months SARS-CoV-2 infection
Source: Infection. 2024 Feb 7;52(4):1269–85. doi: 10.1007/s15010-024-02173-5 (PMC11289012; doi:10.1007/s15010-024-02173-5)
Supplement: Supplementary file 1 — Supplementary file1 (DOCX 615 KB) [file 15010_2024_2173_MOESM1_ESM.docx]

**Supplementary Material**

**Endothelial dysfunction and cardiovascular risk in post-COVID-19 patients after 6- and 12-months SARS-CoV-2** **infection**

Paula Poyatos^1,2^, Neus Luque^1^, Gladis Sabater^1,2^, Saioa Eizaguirre^1,2^, Marc Bonnin^1,2^, Ramon Orriols^1,2,3+^, Olga Tura-Ceide^1,2,3,4+^

^1^Department of Pulmonary Medicine, Dr. Josep Trueta University Hospital de Girona, Santa Caterina Hospital de Salt and the Girona Biomedical Research Institute (IDIBGI), 17190, Girona, Spain.

^2^Department of Medical Sciences, Faculty of Medicine, University of Girona, Girona, Spain. ^3^Biomedical Research Networking Centre on Respiratory Diseases (CIBERES), Madrid, Spain.

^4^Department of Pulmonary Medicine, Servei de Pneumologia, Hospital Clínic-Institut d’Investigacions Biomèdiques August Pi I Sunyer (IDIBAPS), University of Barcelona, Villarroel, 170, 08036 Barcelona, Spain.

^+^**Correspondence**

Olga Tura-Ceide, BSc, PhD, Email: olgaturac@gmail.com, Phone +34-633448238, ORCID ID: 000-0003-4334-9790. Department of Pulmonary Medicine, Dr. Josep Trueta University Hospital de Girona, Santa Caterina Hospital de Salt and the Girona Biomedical Research Institute (IDIBGI), 17190, Girona, Spain.

Ramon Orriols, MBChB, PhD, Email: raorriols.girona.ics@gencat.cat, Phone +34- 972941343, Department of Pulmonary Medicine, Dr. Josep Trueta University Hospital de Girona, Santa Caterina Hospital de Salt and the Girona Biomedical Research Institute (IDIBGI), 17190, Girona, Spain.

**ARTICLE INFORMATION**

Data and supplementary information can be available upon request from the corresponding authors.

**Running title:** Long-term vascular sequelae in COVID-19.

**Table of contents:** Supplementary Tables and Figures.


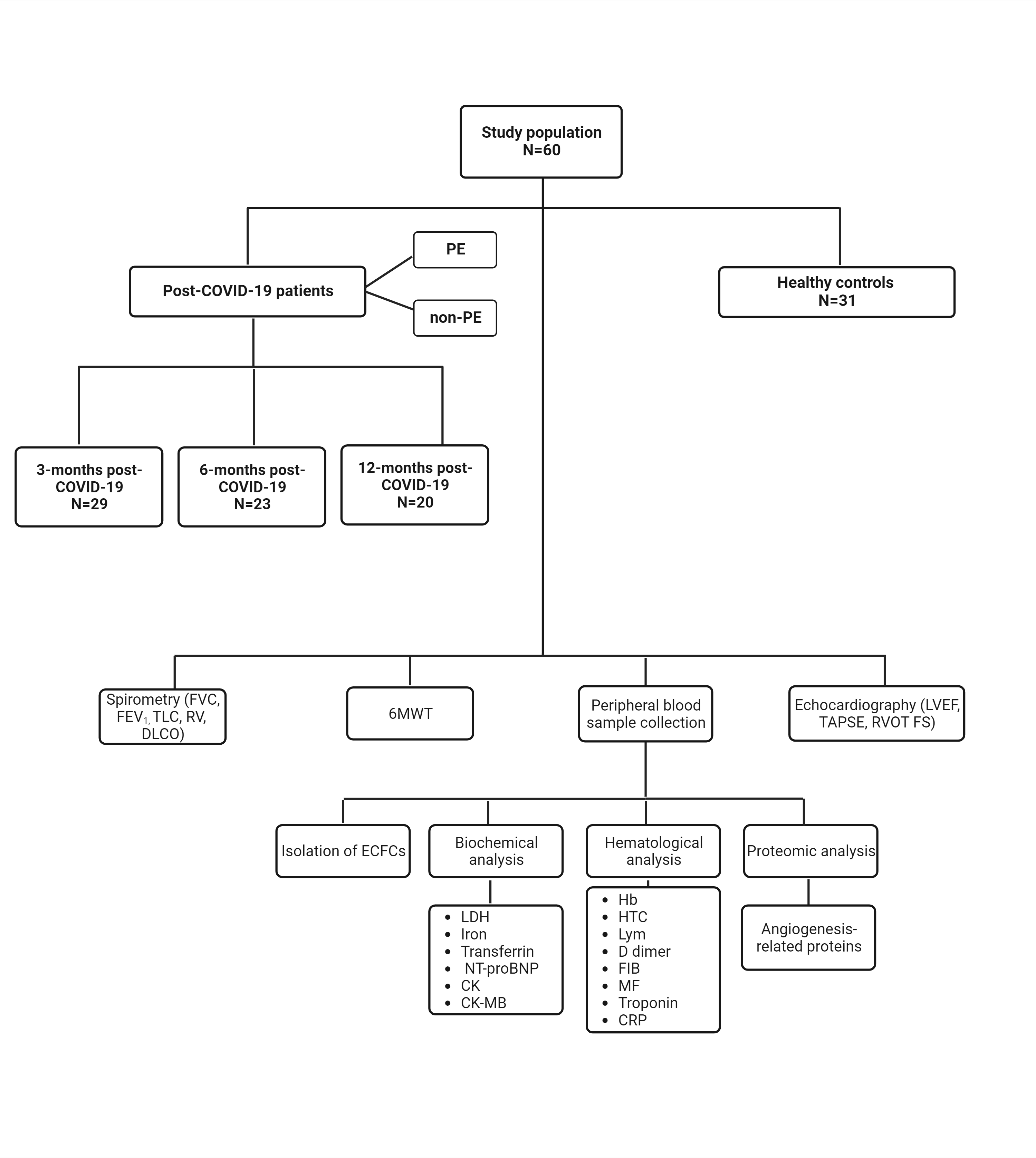


**Supplementary Figure S1.** Flow chart detailing the number of subjects that enrolled in this study and the type of analysis performed. Abbreviations and acronyms: Pulmonary embolism (PE); Forced vital capacity (FVC); Forced expiratory volume (FEV); Total lung capacity (TLC); Residual volume (RV); Carbon monoxide diffusing capacity (DLCO); Six minute walk test (6MWT); Endothelial colony-forming cells (ECFC); Lactate Dehydrogenase (LDH); N-terminal-pro hormone B-type natriuretic peptide (NT-proBNP); Creatinine kinase (CK); Hemoglobin (Hb); Hematocrit (HTC); Lymphocytes (Lym); Dimer-D (DD); Fibrinogen (FIB); Maximum Ferritin (MF); C reactive protein (CRP); Left ventricular ejection fraction (LVEF); Tricuspid annular plane systolic excursion (TAPSE); Right ventricular outflow tract fractional shortening (RVOT FS). Created with BioRender.com.

**A)**

**
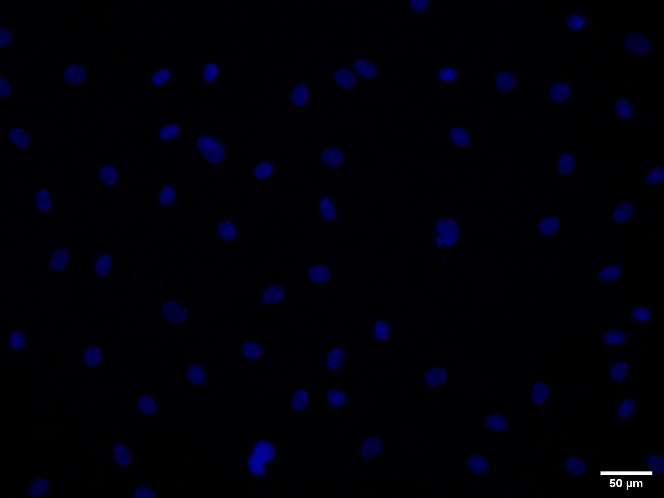

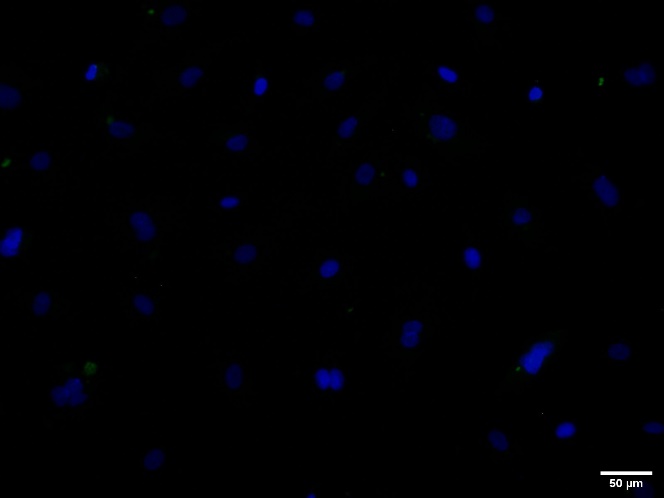
 Post-COVID-19 Healthy controls**

**N- AF488**

**DAPI**

**N- AF488**

**DAPI**

**B)**

|  | **Nucleocapsid (N)** | | | **ORF1** | | | **Rdrp** | | | **TMRSS2** | | |  |
| --- | --- | --- | --- | --- | --- | --- | --- | --- | --- | --- | --- | --- | --- |
|  | **Ct1** | **Ct2** | **Ct3** | **Ct1** | **Ct2** | **Ct3** | **Ct1** | **Ct2** | **Ct3** | **Ct1** | **Ct2** | **Ct3** |  |
| **CL** | UND | UND | UND | UND | UND | UND | UND | UND | UND | 35,473 | 30,525 | 35,569 |  |
|  | UND | UND | UND | UND | UND | UND | UND | 39,864 | 34,796 | 37,345 | 39,110 | UND |  |
|  | UND | UND | UND | UND | UND | UND | UND | UND | UND | 36,411 | 36,459 | 36,483 |  |
|  |  |  |  |  |  |  |  |  |  |  |  |  |  |
| **3 months**  **post-COVID-19** | 36,079 | UND | 34,308 | UND | UND | 35,961 | UND | 38,937 | UND | 39,109 | 36,613 | 35,310 |  |
|  | UND | UND | UND | UND | UND | 37,271 | 34,357 | UND | UND | 37,542 | 35,922 | 37,130 |  |
|  | 37,576 | UND | UND | UND | 38,881 | UND | UND | UND | UND | 35,472 | 35,804 | 35,627 |  |
|  | UND | 35,243 | 35,286 | UND | UND | UND | UND | 39,451 | 36,992 | 39,029 | 36,956 | 38,186 |  |
|  | UND | 33,856 | UND | UND | UND | UND | 33,468 | UND | 33,209 | 36,949 | UND | 39,843 |  |
|  | UND | UND | UND | 39,948 | UND | UND | 33,386 | 39,064 | UND | 38,375 | UND | 34,015 |  |
|  |  |  |  |  |  |  |  |  |  |  |  |  |  |
| **6 months**  **post-COVID-19** | UND | UND | UND | 39,383 | UND | UND | UND | UND | 34,161 | 37,019 | 37,896 | 38,760 |  |
|  | 37,520 | UND | UND | UND | UND | UND | 8,235 | UND | 36,997 | UND | UND | 37,954 |  |
|  | UND | UND | UND | 36,915 | 35,543 | UND | UND | UND | 33,462 | 35,579 | 34,711 | 36,336 |  |
|  | UND | 34,342 | UND | UND | 36,138 | 34,983 | UND | UND | UND | 35,310 | 37,301 | UND |  |
|  | UND | UND | UND | UND | UND | UND | UND | UND | UND | UND | 38,057 | 38,228 |  |
|  | UND | UND | UND | 39,937 | UND | UND | 35,232 | 33,505 | UND | 37,180 | UND | UND |  |

**Supplementary Figure S2. A.** Negative immunostaining for SARS-CoV-2 nucleocapsid (N) protein (green) in ECFCs from post-COVID-19 patients (20x). **B.** NP, ORF1, Rdp and TMRSS2 Ct values of the healthy controls (CL) and post-COVID-19 patients at 3-months and 6 months.

**Supplementary Table S1.** Primers sequence for SARS-CoV-2 detection.

| **Primer Name** | **Forward primer (5'-3')** | **Reverse primer (5'-3')** |
| --- | --- | --- |
| **ORF1ab** | CTAGGACCTCTTTCTGCTCA | ACACTCTCCTAGCACCATCA |
| **RdRp** | CATCTCACTTGCTGGTTCCT | CCTTAATAGTCCTCACTTCTCTC |
| **N** | CCTCTTCTCGTTCCTCATCA | CCTGGTCCCCAAAATTTCCT |

**Supplementary Table S2.** Correlation coefficients (r) for the relationship between troponin levels and NT-proBNP levels in COVID-19 patients at 3, 6 and 12-months after infection. Spearman rank correlation, **P <0.01.

| **Variables** | **3 months post-COVID-19, n=20** | | **6 months post-COVID-19, n=20** | | **12 months post-COVID-19, n=20** | |
| --- | --- | --- | --- | --- | --- | --- |
|  | **Correlation coefficient (r)** | **P-value** | **Correlation coefficient (r)** | **P-value** | **Correlation coefficient (r)** | **P-value** |
| **NT-proBNP (pg/mL)** | 0,12 | 0,25 | **0,65** | **0,003**** | 0,30 | 0,21 |

**Abbreviations and acronyms:** N-terminal-pro hormone B-type natriuretic peptide (NT-proBNP).

**Supplementary Table S3. Long COVID incidence. A.** Frequency of Long COVID of 3, 6 and 12-months post-COVID-19 patients, with or w/o pulmonary embolism (PE), Chi-square test, P > 0.05. **B.** Frequency of long COVID symptoms in 3, 6 and 12-months post-COVID-19 patients, Chi-square test, P > 0.05.

| **Variables** | **3 months**  **post-COVID-19,**  **n=29** | | **6 months**  **post-COVID-19,**  **n=23** | | **12 months**  **post-COVID-19,**  **n=20** | | **P-value** |
| --- | --- | --- | --- | --- | --- | --- | --- |
| 1. **Long COVID (%)** | 19 (67,9%) | 14 (73,7%) **w/o** **PE** | 12 (52,2%) | 7 (46,7%) **w/o** **PE** | 8 (42,1%) | 5 (41,7%) **w/o** **PE** | ns |
|  |  | 5 (55,6%) **with PE** |  | 5 (62,5%) **with PE** |  | 3 (42,9%) **with PE** |  |
| 1. **Long COVID Symptoms** | | | | | | | |
| **Fatigue (%)** | 7 (25,0%) | | 4 (17,4%) | | 1 (5,26%) | | ns |
| **Low mobility (%)** | 2 (7,14%) | | 2 (8,70%) | | 0 (0,00%) | | ns |
| **Constipation (%)** | 4 (14,3%) | | 1 (4,35%) | | 0 (0,00%) | | ns |
| **Joint pain (%)** | 2 (7,14%) | | 0 (0,00%) | | 2 (10,5%) | | ns |
| **Chest pain (%)** | 4 (14,3%) | | 1 (4,35%) | | 1 (5,26%) | | ns |
| **Haedache (%)** | 0 (0,00%) | | 0 (0,00%) | | 1 (5,26%) | | ns |
| **Tinnitus (%)** | 1 (3,57%) | | 0 (0,00%) | | 1 (5,26%) | | ns |
| **Myalgia (%)** | 0 (0,00%) | | 0 (0,00%) | | 2 (10,5%) | | ns |
| **Hoarseness (%)** | 1 (3,57%) | | 1 (4,35%) | | 0 (0,00%) | | ns |
| **Edema (%)** | 3 (10,7%) | | 0 (0,00%) | | 0 (0,00%) | | ns |
| **Abdominal pain (%)** | 0 (0,00%) | | 1 (4,35%) | | 1 (5,26%) | | ns |
| **Skin rashes (%)** | 0 (0,00%) | | 0 (0,00%) | | 1 (5,26%) | | ns |
| **Memory loss (%)** | 0 (0,00%) | | 1 (4,35%) | | 2 (10,5%) | | ns |
| **Difficulty concentrating (%)** | 1 (3,57%) | | 0 (0,00%) | | 2 (10,5%) | | ns |
| **Insomnia (%)** | 1 (3,57%) | | 1 (4,35%) | | 0 (0,00%) | | ns |
| **Paresthesia (%)** | 3 (10,7%) | | 1 (4,35%) | | 1 (5,26%) | | ns |
| **Myeloradiculoneuritis (%)** | 1 (3,57%) | | 1 (4,35%) | | 0 (0,00%) | | ns |
| **Anxiety (%)** | 1 (3,57%) | | 2 (8,70%) | | 0 (0,00%) | | ns |
| **Nasal packing (%)** | 0 (0,00%) | | 1 (4,35%) | | 0 (0,00%) | | ns |
| **Disgeusia (%)** | 2 (7,14%) | | 0 (0,00%) | | 0 (0,00%) | | ns |
| **Anosmia (%)** | 4 (14,3%) | | 0 (0,00%) | | 0 (0,00%) | | ns |
| **Dyspnea (%)** | 1 (16,7%) | | 4 (66,7%) | | 3 (42,9%) | | ns |
| **Cough (%)** | 0 (0,00%) | | 0 (0,00%) | | 0 (0,00%) | | ns |
| **Expectoration(%)** | 1 (3,57%) | | 0 (0,00%) | | 0 (0,00%) | | ns |
| **Thrombosis (%)** | 0 (0,00%) | | 1 (4,35%) | | 0 (0,00%) | | ns |
| **Heart palpitations (%)** | 0 (0,00%) | | 0 (0,00%) | | 1 (5,26%) | | ns |

**Abbreviations and acronyms:** Healthy control (CL); Pulmonary embolism (PE).

**Supplementary Table S4.** Correlation coefficients (r) for the relationship between the presence of Long-COVID and the different variables analyzed in COVID-19 patients at 3, 6 and 12-months after infection. Spearman rank correlation, *P <0.05.

| **Variables** | **3 months post-COVID-19, n=29** | | **6 months post-COVID-19,**  **n=23** | | **12 months post-COVID-19,**  **n=20** | |
| --- | --- | --- | --- | --- | --- | --- |
|  | **Correlation coefficient (r)** | **P-value** | **Correlation coefficient (r)** | **P-value** | **Correlation coefficient (r)** | **P-value** |
| **Age, years** | -0,04 | 0,83 | -0,09 | 0,70 | -0,26 | 0.28 |
| **Female sex n** | 0,12 | 0,54 | **0,44** | **0,04*** | 0,40 | 0,09 |
| **BMI (Kg/m^2^)** | 0,02 | 0,90 | -0,32 | 0,14 | -0,39 | 0,09 |
| **AHT** | 0,03 | 0,89 | -0,30 | 0,16 | -0,17 | 0,49 |
| **DM** | 0,19 | 0,33 | 0,30 | 0,17 | -0,29 | 0,22 |
| **DLP** | 0,06 | 0,75 | -0,02 | 0,93 | -0,37 | 0,12 |
| **Number of ECFC colonies** | 0,24 | 0,22 | -0,05 | 0,81 | 0,12 | 0,63 |
| **Appearance of ECFC colonies** | -0,12 | 0,54 | 0,15 | 0,51 | 0,29 | 0,22 |
| **Time for ECFC**  **to appear (days)** | UND | UND | 0,16 | 0,50 | 0,01 | 0,98 |
| **FVC (%)** | 0,15 | 0,48 | -0,62 | 0,14 | 0,11 | 0,88 |
| **FEV_1_ (%)** | -0,06 | 0,77 | -0,39 | 0,39 | -0,28 | 0,52 |
| **FEV_1_/FVC (%)** | -0,31 | 0,13 | -0,17 | 0,79 | -0,62 | 0,14 |
| **RV (L)** | 0,29 | 0,17 | UND | UND | UND | UND |
| **DLCO (%)** | 0,28 | 0,15 | -0,23 | 0,61 | -0,45 | 0,27 |
| **6MWT (m)** | 0,23 | 0,25 | -0,87 | 0,67 | 0,17 | 0,79 |
| **Hb (g/dL)** | 0,15 | 0,52 | **-0,43** | **0,049*** | -0,14 | 0,58 |
| **HTC (%)** | 0,29 | 0,21 | -0,37 | 0,09 | -0,02 | 0,94 |
| **Lym (K/mcL)** | -0,12 | 0,61 | 0,14 | 0,54 | 0,23 | 0,34 |
| **LDH (mg/dL)** | 0,02 | 0,94 | -0,04 | 0,87 | 0,02 | 0,94 |
| **MF (ng/mL)** | **0,48** | **0,03*** | -0,22 | 0,34 | 0,29 | 0,23 |
| **CRP (mg/dL)** | -0,03 | 0,89 | -0,02 | 0,95 | 0,02 | 0,94 |
| **Troponin (ng/L)** | -0,04 | 0,86 | -0,18 | 0,43 | -0,12 | 0,63 |
| **Positive DD (%)** | UND | UND | -0,29 | 0,80 | 0,87 | 0,67 |
| **FIB (mg/dL)** | 0,20 | 0,38 | 0,41 | 0,06 | 0,16 | 0,52 |

**Abbreviations and acronyms:** Healthy control (CL); Body mass index (BMI); Arterial hypertension (AHT); Diabetes mellitus (DM); Dyslipidemia (DLP); Forced vital capacity (FVC); Forced expiratory volume (FEV); Total lung capacity (TLC); Residual volume (RV); Carbon monoxide diffusing capacity (DLCO); Six-minute walk test (6MWT); Hemoglobin (Hb); Hematocrit (HTC); Lymphocytes (Lym); Lactate Dehydrogenase (LDH); Maximum Ferritin (MF); C reactive protein (CRP); Dimer-D (DD); Fibrinogen (FIB).

**Supplementary Table S5.** Clinical characteristics of post-COVID-19 patients at different time points after overcoming COVID-19 (3,6 and 12 months). Chi squared test for categorical variables *P < 0.05.

| **Variables** | **CL,**  **n=31** | **3 months**  **post-COVID-19,**  **n=29** | **6 months**  **post-COVID-19,**  **n=23** | **12 months**  **post-COVID-19,**  **n=20** | **P-value**  **(COVID-19 vs CL)** |
| --- | --- | --- | --- | --- | --- |
| **History of myocardial infarction (%)** | 0 (0%) | 4 (13,8%)* | 3 (13,0%)* | 2 (10,0%) | P<0.05 |
| **History of asthma (%)** | 0 (0%) | 1 (3,45%) | 1 (4,35%) | 1 (5,00%) | ns |
| **History of COPD (%)** | 0 (0%) | 4 (13,8%)* | 2 (10,0%) | 1 (5,00%) | P<0.05 |
| **Cardiac disease (%)** | 1 (3,23%) | 6 (20,7 %)* | 4 (17,4%) | 5 (25,0%)* | P<0.05 |
| **AHT (%)** | 7 (22,6%) | 13 (44,8%) | 11 (47,8%) | 9 (45,0%) | ns |
| **DM (%)** | 0 (0,00%) | 3 (10,3%) | 2 (8,70%) | 2 (10,0%) | ns |
| **DLP (%)** | 2 (6,45%) | 5 (17,2%) | 4 (17,4%) | 3 (15,0%) | ns |
| **Auto-immune disease (%)** | 1 (3,23%) | 1 (3,45%) | 1 (4,35%) | 1 (5,00%) | ns |

**Abbreviations and acronyms**: Healthy control (CL); Chronic obstructive pulmonary disease (COPD); Arterial hypertension (AHT); Diabetes mellitus (DM); Dyslipidemia (DLP).

**Supplementary Table S6.** CK and CK-MB levels of healthy controls (CL) and 3,6 and 12-months post-COVID-19 patients. Values expressed as mean ± SD. Unpaired t test for parametric tests, Mann-Whitney test for non-parametric tests P > 0.05.

| **Variables** | **CL** | **3 months**  **post-COVID-19** | **6 months**  **post-COVID-19** | **12 months**  **post-COVID-19** | **P-value**  **(COVID-19 vs CL)** |
| --- | --- | --- | --- | --- | --- |
| **CK (U/L)** | ND | 93,2 ± 61,4 | 144 ± 119 | 126 ± 59,1 | ND |
| **CK-MB (U/L)** | ND | 21,3 ± 8,84 | 23,0 ± 3,58 | 18,3 ± 4,11 | ND |

**Abbreviations and acronyms**: Healthy control (CL); Creatinine kinase (CK).

**Supplementary Table S7.** LVEF, TAPSE and RVOT FS values of healthy controls (CL) and 3 and 6-12-months post-COVID-19 patients, with or w/o pulmonary embolism (PE). Values expressed as mean ± SD. Unpaired t test for parametric tests and Mann-Whitney test for non-parametric tests, P > 0.05.

| **Variables** | **3 months post-COVID-19** | | **6-12 months post-COVID-19** | | **P-value** |
| --- | --- | --- | --- | --- | --- |
| **LVEF (%)** | 63,0 ± 3,08  **n=12** | | 58,4 ± 8,7  **n=12** | | ns |
| **TAPSE (mm)** | 23,8 ± 3,17  **n=16** | | 20,8 ± 5,05  **n=13** | | ns |
| **RVOT FS (%)** | 46,9 ± 4,03  **n=8** | | 47,1 ± 7,62  **n=11** | | ns |
| **Variables** | **3 months post-COVID-19 w/o** **PE** | **3 months post-COVID-19 with PE** | **6-12 months post-COVID-19 w/o** **PE** | **6-12 months post-COVID-19 with PE** | **P-value (COVID-19 with PE vs w/o PE)** |
| **LVEF (%)** | 62,6 ± 3,26 | 63,6 ± 3,05 | 57,8 ± 12,8 | 59,0 ± 1,16 | ns |
| **TAPSE (mm)** | 24,7 ± 2,56 | 23,0 ± 3,54 | 23,5 ± 1,87 | 18,4 ± 5,86 | ns |
| **RVOT FS (%)** | 45,2 ± 3,08 | 48,0 ± 4,47 | 50,6 ± 3,28 | 45,1 ± 8,86 | ns |

**Abbreviations and acronyms:** Pulmonary embolism (PE); Left ventricular ejection fraction (LVEF); Tricuspid annular plane systolic excursion (TAPSE); Right ventricular outflow tract fractional shortening (RVOT FS).
